# A Special Relationship—Aspects of Human–Animal Interaction in Birds of Prey, Brown Bears, Beavers, and Elk in Prehistoric Europe

**DOI:** 10.3390/ani14030417

**Published:** 2024-01-27

**Authors:** Ulrich Schmölcke, Oliver Grimm

**Affiliations:** Leibniz-Zentrum für Archäologie, Centre for Baltic and Scandinavian Archaeology (LEIZA-ZBSA), Schloss Gottorf, D-24837 Schleswig, Germany; oliver.grimm@leiza.de

**Keywords:** human–animal studies, human–animal relationships, long-term consideration, social zooarchaeology, perception of animals, archaeozoology, zooarchaeology, archaeology, cultural history, falconry, rituals

## Abstract

**Simple Summary:**

This paper looks at past relationships between humans and certain wild animal species, that is, raptors, bears, beavers, and elks. As we have observed, it was the behaviour of animals that caught the attention of humans, such as spectacular flights and hunting by raptors, the similarities in bear and human behaviour, the fascinating impressiveness and charisma of elks, and the landscape-shaping by enigmatic beavers. All these animals have special meanings to humans far beyond their economic significance. Wild goshawks, falcons, and other raptor species have acted as trained hunting companions, but they were never domesticated and had to be treated with care. As for bear, beaver, and elk, there were often complex rituals before, during, and after the killing, because there was no clear separation between humans and animals. The animals were not just prey but equal beings and other-than-human persons. However, there were also changes over time in the human–animal relationship, such as the advent of the farming way of life with a preference for domesticated animals, as well as the arrival of Christianity, by which animals became second to human.

**Abstract:**

Humans have developed a special relationship with some animal species throughout history, even though these animals were never domesticated. Based on raptors, bears, beavers, and elks, the question of whether there are similarities between the perception of these animals that triggered a special kind of fascination in humans and how the relationship between humans and these animals changed between Mesolithic age and medieval times is addressed. As we demonstrate, the categorical antagonism between ‘animal’ and ‘human’ is a concept that saw different kinds of influence, from the advent of sedentarism and husbandry to Christianity and from philosophical thinking in Classical Antiquity and the Period of Enlightenment. In prehistory and early history, we find different, opposing world views across time, cultures, and periods. Differences between animals and humans have been considered as fluid, and humans have had to engage with animals and their needs. The well-known and famous ‘bear ceremonies’ attested to different peoples and times were not unique, but were a part of belief systems that also included other animal species. Among the considered animals, certain raptor species attracted the attention of humans who tried to establish contact with them, as companions, whereas bears were almost ‘disguised humans’ due to all their similarities with humans, but they were also tabooed beings whose real names had to be avoided.

## 1. Introduction

Human–animal studies have their point of departure in the Animal Rights Movement of the 1970s, as an alternative view that distances itself from the prevailing, purely utilitarian, human perception of animals that can also be understood as ‘othering’ [1,2]. This rethinking of the human–animal relationship leads to a critical view not only of the treatment of animals in modern times but also the underlying historical elements in Western thought, such as the demonstration of animals as non-reasonable and thus second to the humans expressed, inter alia, by Aristotle in Classical Antiquity and by René Descartes in the Period of Enlightenment (‘I think therefore I am’). However, in this respect, Christianity also requires a mention regarding this well-known demand: ‘Be fruitful and multiply and fill the earth and subdue it, and have dominion over the fish of the sea and over the birds of the heavens and over every living thing that moves on the earth’ (Genesis 1:28) [1].

However, the use of the term ‘animal’ as an antithesis to ‘human’ undercuts fundamental evolutionary facts, and ‘a look at other times and other cultures reveals that such a boundary is actually a special case. Some peoples and cultures are not aware of any boundary, while for others it is fluid’ [2,3]. In the meantime, an alternative view of animals and humans is increasingly assumed by both the public and academia.

Hallmarks of academic recognition of the theme have been, inter alia, the establishment of the periodicals *Anthrozoös. A Multidisciplinary Journal of the Interactions of People and Animals* in 1987 and *Society & Animals* in 1993 [1,2]. The first publications in this field were authored by philosophers [4,5] before representatives of the Humanities and Social Sciences took a broader interest, often with a focus on recent times due to the obvious number and significance of written and pictorial testimonies [1,2].

Archaeo(zoo)logy entered the discussion on human–animal studies only later, as the so-called Social Zooarchaeology, which was advocated by different researchers around a decade ago [6,7]. However, only in exceptional cases have animal remains from archaeological excavations been evaluated since then, beyond categories such as protein, calorie, and material use, and with a new focus on emotional relationships, including the role of animals in symbolism, rituals, and cults, or as status indicators and symbols.

Human–animal studies in archaeo(zoo)logy still seem to be rather rare, which is surprising given the importance of this branch for the reconstruction of past worldviews [8,9,10,11,12]. As was demonstrated in former studies [13,14,15], the consideration of raptor–human and bear–human relationships benefits from a long-term and interdisciplinary perspective, which is set in motion in archaeo(zoo)logy itself when the needed time-depth is added to the discourse that is required for any broader look on human–animal relations. The very lives of the animals themselves need consideration, too, and here biology plays a key role in the mentioned books.

In this paper, we would like to show the potential of human–animal studies in archaeo(zoo)logy with a look at chosen wild animal species; in a second step, source materials from other academic branches will be included, interdisciplinarily, but practical knowledge will also be included, transdiciplinarily. It is precisely the diversity of the chosen animals that may lead to the recognition of patterns, trends, and transformations in the relationship between those species and humans. It is also a search for the roots and effects of the dichotomy between ‘animal’ and ‘human’ that is so strict today. Of particular importance is the long-term perspective (longue durée), since only this will allow us to see changes in attitude, according to the historian Ferdinand Braudel from the second generation of the French Annales school, who established this type of perspective [16,17]. Archaeo(zoo)logically, comparisons of animal–human relationships in hunter–gatherer vs. farmer societies are as meaningful as the study of changes in belief or increasing social complexity.

For the present study, certain species were selected, often based on our own preliminary studies, as being particularly exemplary for investigating prehistoric relationships between humans and animals. Humans shared the same habitat with moose, beavers, and bears for many millennia. All of the selected species were widespread in the study area, i.e., that everyone knew. At the same time, each of these species has distinctive behavioural characteristics that make it inevitable that (prehistoric) humans were intensively involved with them. Birds of prey are also conspicuous and possibly significant because of their behaviour. They gained human attention after people learned to tame them and go hunting with them. This resulted in a really unique animal–human relationship.

## 2. Materials and Methods

This study presents and compares human–animal relationships using four very different species (or species groups) in prehistoric and early historic Europe. The geographic focus is on Central and Northern Europe. We divided our study area into three parts: Fennoscandia (FS; Norway, Sweden, and Finland), western North Central Europe (western CE; Czech Republic, Germany, and Denmark), and eastern Central Europe (eastern CE; Estonia, Latvia, Lithuania, and Poland). The archaeozoological data for this study are gathered from the huge data collection “The Holocene History of the European Vertebrate Fauna”, which was built up under the leadership of Angela von den Driesch (Ludwig Maximilians University, Munich), Norbert Benecke (German Archaeological Institute, Berlin), and Dirk Heinrich (Christian Albrechts University, Kiel) in the 1990s [18].

With respect to FS, we integrated the archaeozoological data from 465 archaeological sites with altogether 60,000 remains from wild animals (NISP: Number of Identified Specimen; Table 1). For eastern CE, we include 578 sites with a total NISP of about 154,000 (Table 2), and for western CE, there are 1511 sites with NISP of 160,000 (Table 3). In FS, 58% of the sites taken under consideration are from former settlement contexts, 27% from different kinds of human graves (the rest: other features). In the eastern CE, the proportions of these two groups of features are 91% to 5%; in the western CE the proportions are 69% to 22%.

In addition to the proportions in the total NISP of the species analysed here, constancy is a valuable measurement used in this paper. Constancy means the presence of a species in particular sites or periods [19,20,21]. For constancy, the absolute number of individuals from this species at one site or in one period is not important, and herein constancy has some similarity with the biogeographical concept of nestedness [22]. Constancy compares the presence or absence of species and is not related to the type of skeletal element, degree of fragmentation, or modifications the excavated elements went through (cut marks, gnawing traces, and perforations). In contrast, the proportion of a species in the NISP reflects the (food) economic importance of the species and thus indirectly also its frequency.

Prior to historic times with cultures that have left written sources, it is difficult to reconstruct the human perception of other animal species and the general relationships between birds of prey, brown bears, beavers, or elk, on the one hand, and humans, on the other, and vice versa. Working with archaeozoological data alone would not be successful. Here, we include data from ethnoarchaeology, ethnology, archaeology, and wildlife biology as well as written sources and imagery. Only if such historical sources complement archaeozoological records will our knowledge about (pre)historical human–animal relationships have a proper foundation.

The main task of the present study is to create an overview of the development of selected human–animal relationships in the area under consideration through time. Changes and developments in the human perception of the selected species are always related to cultural customs and practices, so it makes sense to use a chronological timeline based on the main cultural periods for the diachronic structure. It is clear that such a structure simplifies archaeological insights and units, but it is also suitable in the context of the questions to be handled here. Thus, the postglacial period will be divided into the following stages: Mesolithic age (9600–4000 BCE), Neolithic age (4000–1800 BCE), Bronze Age (1800–500 BCE), Pre-Roman Iron Age (500–1 BCE), Roman Iron Age (0–500 CE), early medieval period (500–1050 CE), and late medieval period (1050–1500 CE). We are aware that this categorization is not common in the entire study area, and we are also aware that there were sometimes strong discrepancies between different regions within a time slice. However, we believe that this categorization is nonetheless useful in the context of this study, which covers a wide range of time periods and cultural areas.

## 3. Results

### 3.1. Birds of Prey

Our evaluations show that birds of prey are detected in archaeological assemblages with less constancy than the mammalian species discussed below. The constancy with which even the most frequent raptor species are found at archaeological sites of a particular epoch is often only 1–2%. White-tailed eagles (*Haliaeetus albicilla*) are usually the most abundantly recorded raptor species in all three study regions and in all periods investigated. This huge bird, which lives near all large bodies of water, was an attractive hunting bird of prey. However, the results of our study show striking developments in space and time concerning the constancy of white-tailed eagles and other birds of prey.

At Mesolithic forager stations, bones of white-tailed eagles are found with high constancy throughout the study area. In western CE and eastern CE, they are recorded at every fourth to fifth site (a constancy of 23% and 20%, respectively), which is an enormously high value for birds of prey (Table 4 and Table 5). White-tailed eagles are also the raptor species with the highest constancy in FS, although the value here is significantly lower, at 5% (Table 6). Overall, raptor bones are much rarer at Stone Age sites in FS than in western CE. The data basis for eastern CE is considerably smaller, but in eastern CE there are more similarities to western CE than to FS.

Records of birds of prey in burial contexts are rare. Only at the cemetery island of Oleniy Ostrov, located on Lake Onega, Karelia, is it evident that the osprey (*Pandion haliaetus*) played a special role, at least in the regional ideology, in about 6400 BCE [23,24]. In one case, two legs of an osprey were deposited by the body of an elderly man, and, in another case, parts of a wing were deposited by the body of a child. In the context of our study, it is particularly noteworthy that, in addition to the remains of this bird of prey, the same graves also contained tooth pendants made of elk and beaver incisors. Bones from the white-tailed eagle were found at Oleniy, too, but none of these could be related to a particular grave [23]. Additionally, at the Neolithic burial site of Tamula (Estonia), in about 4200 BCE, an adult male was buried together with parts of a golden eagle [23,25]. All the other Stone Age remains of raptor species from the study area derive from settlement contexts.

It is noticeable in all study regions that the evidence of birds of prey becomes rarer with the beginning of the Neolithic age. In FS, this trend continues into the Iron Age, in eastern CE even to Roman Period, when birds of prey can only be detected sporadically. The few records from the Roman Period come almost exclusively from eagles—white-tailed as well as golden eagles (*Aquila chrysaetos*). The development in western CE is somewhat different. Here, the numbers and frequency of birds of prey in the archaeological material increase again in the Iron Age. The constancy values are still much lower than for bear, elk or beaver, but at least an increase in the relative number of records can be observed for white-tailed eagle, golden eagle, goshawk (*Accipiter gentilis*), and red kite (*Milvus milvus*), as compared to earlier epochs.

From the Iron Age to the late medieval period, the relative number of records increase slowly but steadily for many raptor species in western CE, albeit at a low level. This applies, in particular, to the white-tailed eagle, goshawk, sparrow hawk (*Accipiter nisus*), common kestrel (*Falco tinnunculus*), peregrine falcon (*Falco peregrinus*), red kite, and common buzzard (*Buteo buteo*). In the Roman period and early medieval, the white-tailed eagle—mostly, again, the raptor species with the highest consistency—can be found at every 20th archaeological site, and even at the late medieval sites at every eleventh site. In the late medieval sites, goshawks are found even more regularly than white-tailed eagles (a constancy of 11%).

In eastern CE, the development is very similar. In no time slice is the number of recorded species of birds of prey higher (this is, of course, also due to the significantly larger number of sites from the late medieval period, so it is also a statistical effect). From early medieval times, goshawks replaced white-tailed eagles as the most frequently detected species and can be found at one in seven sites (a constancy of 14%)—this is a very high value for birds of prey. Other species such as the sparrow hawk, common kestrel, and common buzzard now also reach peak values.

The development in FS was noticeably different to the other regions, at least on a completely different level. Birds of prey also played a minor role here until the Roman Period, but this changed suddenly and completely in the early medieval period. Bones of hawks can now suddenly be found at almost three-quarters of all sites (a constancy of 72%). This is the highest value of all species discussed in this paper. Sparrow hawks and peregrine falcons also reach stable constancy values of over 10%. In late medieval times, the hawk’s constancy declined to a—still high—25%. Presently, the white-tailed eagle has reached a maximum value with evidence at 27% of the archaeological sites. As we will see, they are found in the late medieval period with a much higher degree of continuity than, for example, bears and beavers.

In order to classify these developments, the archaeological background of the finds must be taken into account. For earlier periods, raptors have come to light only sporadically in burials (for Sweden [26]) but, for the middle and later part of the first millennium AD, mostly the goshawk but also the sparrow-hawk, peregrine falcon, and, rarely, gyrfalcon (*Falco rusticolus*) are now found in dozens of burials [27,28]. This is the case for parts of FE (Norway and Sweden) and north-western CE (northern Germany), whereas no such finds have come to light from Denmark, England, and the entire Slavic area east of the Elbe River. As we will discuss later, Lithuania is somehow special due to its records.

Sweden is outstanding with c. 45 graves, mostly cremation, which were directly covered by mounds after the funeral pyre had burnt down [27,29]. The carefully excavated burial at Rickeby, to the north of Stockholm, demonstrates that entire animals were deposited: one horse, several dogs, several raptors, and other birds. The respective burials in Sweden are usually above average in social standing, and a substantial number of these are men with weapons and helmets, which could be interpreted as members of retinues.

There are also instances beyond Sweden that deserve a mention. Goshawk bones have come to light during the re-analysis of animal remains found in the southeast Norwegian ‘royal’ Viking Age ship burial at Gokstad, dated to around 900 CE, and is the only Norwegian find to that effect so far [28,30]. When it comes to western CE, only a handful of inhumation graves with goshawks are known, the oldest of which is that of a wealthy woman in Quedlinburg (c. 500 CE), alongside likewise wealthy men with weapons in Eschwege-Niederhone and Alach [31,32]. The latter three are the so-called ‘Gründergräber’, the earliest and at the same time richest in the given cemeteries, which is a hallmark of a new leading family.

The somewhat enigmatic late Vendel Age ship finds at Salme, outer Estonia (c. 700 CE), cannot be elaborated upon here [33]. The slain warriors in the vessels originate from Sweden, but why did they bring a number of raptors with them? It is also worth a mention that, according to Wigand von Marburg, a scribe of the Teutonic Order, a Duke of the Grand Duchy of Lithuania received many animals for his burial in late 1300 CE, including raptors [34]. No such thing has been found in burials so far.

Parallel to the appearance of particular raptors in burials, the same bird species also came from settlement contexts in remarkable numbers, although, in this case, skeletons of birds carry more evidential value than single bones (see [35], as an introduction). Remarkably, the number of Swedish sites with such bones amounts to 40 in the period from c. 600 to 1500 CE and, notably, these are not rural but ‘special sites’: royal seats, trading places, and cities [27]. Also, one Norwegian royal seat, from early post-1000 CE, in the very south-east of the country (present-day Kungahälla, situated in Bohuslän along the west coast of Sweden) has yielded a number of raptor bones, again mainly goshawks [36]. The same pattern can be observed in Sweden; the bones of goshawk (but also the sparrow-hawk and peregrine falcon) from special sites can be encountered for in western CE, mostly for fortifications as seats of noble persons in the period post-1000 CE [37]. As regards raptors from trading sites, such as Groß Strömkendorf (Reric) and also Birka in Sweden, one may argue that these birds were trading goods, as is known from younger periods, whereas for finds in (pre)urban contexts, a close look on the area of provenance is needed; do they originate from ordinary living quarters or other parts of the city [35]?

Denmark and the west Slavic area in the north of western CE become visible not by raptor species in graves but again by finds from settlement contexts. For instance, partial raptor skeletons originate from Schleswig (layers from the 11th to 14th century CE), with the Danish royal seat nearby. The houses themselves at Schleswig are ordinary in size; however, they stand out in two respects, namely silk finds and actual bird skeletons, which are a rare find [38]. Totally unique with its minimum number of 41 raptors from several species, including a number of partial skeletons, is the West-Slavic seat of power in Starigard-Oldenburg, with its period of use in late pre-1000 CE [39,40]. The situation is more complex later on with a repeated shift in political control between West-Slavic rulers and church representatives (bishops), which has to be omitted here. Raptor bones were also recorded in Mikulčice, the seat of power of the short-lived Great Moravia, mainly in the ninth century CE [41,42].

Apart from bones, there is a second archaeological source material, the visual one, that needs a mention; this is represented by riders with raptors on their fist. Recently, a fibula with such an image has come to light in the burial of a woman, dated to the seventh century CE and above average in furnishings, in the cemetery in Münstermaifeld, western Germany [43]. This find is more or less contemporary with the aforementioned burials from Quedlinburg, Alach, and Eschwege-Niederhone (all western CE). Another similar record derives from a wealthy burial in Stare Mesto, one more seat of power of Great Moravia [44]. Depictions of riders with raptors on their fist are also known from FS, for the Viking Age and the following centuries [45,46]. Outside our main area of concern, but important, such imagery is encountered in mosaics for areas that saw a Germanic intrusion during the Migration Period (these mosaics can probably be ascribed to the Vandals in Tunisia and the Westgoths on the Iberian Peninsula, whereas authors from Greek and Roman antiquity did not know about this kind of hunting [47]).

There is yet another, third, source material that comes to mind in the present context. Very rarely, birds, bones, or skeletons have been found together with either small metal bells or gloves and hoods of organic materials. Of particular importance is a complete skeleton of a gyrfalcon (here, an exotic species; cf. [48]), found together with a bell at the medieval French royal seat, which was unearthed beneath the present Louvre [49]. But, notably, these finds do not speak for themselves, they can only be understood in a broader context.

At the same time, eagle remains were sometimes found in large quantities in settlement contexts, e.g., in the aforementioned Hedeby (western CE; 9th–11th century CE [50]) or Klaipėda in Lithuania (eastern CE; 13th–14th century CE [51]. In both cases, the eagle bones were found in common household rubbish. Smaller numbers of such finds are common throughout the medieval period.

### 3.2. Brown Bear

**Figure 1:**
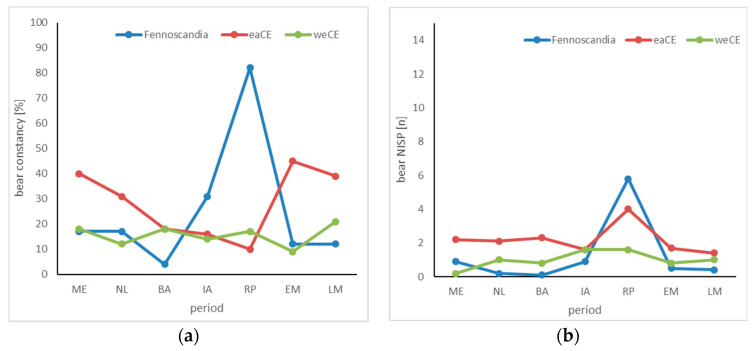
Brown bear (*Ursus arctos*) at the archaeological sites in Fennoscandia, eastern Central Europe (eaCE), and western Central Europe (weCE) in different periods. ME: Mesolithic, NL: Neolithic, BA: Bronze Age, IA: Iron Age, RP: Roman period, EM: early medieval, and LM: late medieval. (**a**) constancy; (**b**) proportions of the number of identified wild mammal specimens.

The constancy of bear remains at archaeological sites is the greatest in eastern CE during almost all periods (Figure 1a). Two trends can be observed there: After the Mesolithic age, the consistency of bear remains decreases slowly but continuously from 40% to 10%, with its lowest value during the Roman period. Afterwards, in medieval times, there is a renewed, sharp increase to values that are still above those of the Mesolithic age. Exactly opposite is the development in FS. Coming from relatively low values of a maximum of 17%, the constancy of bear remains here reaches high values in the Iron Age (31%) and then an extraordinarily high value of 82% during the Roman period; afterwards, the values drop back to the earlier, relatively low level. In western CE, in contrast, there are no significant variations in the constancy of bear findings over the investigated millennia. The values fluctuated by 16% over the entire period of the study.

The proportion of bears in the NISP of wild animals is always low and mostly between 1 and 2% (Figure 1b); however, it shows maximum values during the Roman period in all three regions studied. While this peak is hardly pronounced in western CE, it is very clear in eastern CE and FS. Here, 4% (eastern CE) and 6% (FS) of the wild animal bones originate from bears at this time. After the Roman period, the proportions return to the earlier level.

As was the case with the aforementioned raptors, bear remains used to be a rare grave find for earlier times (see [52] for Central Europe), but they became very numerous in parts of the first millennium AD, again with a particular emphasis on Sweden [53]. Burials with claws, and rarely with teeth or actual skins, are recorded as a particular burial rite for substantial parts of the Iron Age in both FS (foremost in Sweden and less so in Norway) and northern western CE (Denmark and Germany, but hardly at all in the Slavic region east of the Elbe river [54,55,56,57,58]. Two focal ranges can be noticed; one being the transition from the late pre-Roman to the Roman Iron Age and thus c. 2000 years ago, and the other for the middle of the first millennium CE. As a major rule, it seems that bear claws were added to the funeral pyre and later placed in the urns of the deceased. The main part of the burials has been granted one to five claws, which, by the mere number, may indicate a paw rather than a skin. Generally, men and women received such claws in cremation burials, which range from poor to wealthy but, as an exception from the rule, a continental group, again around 2000 years of age, consists of cremated men that are often richly furnished [59]. Yet another exception are cremation burials in Funen, Denmark, which date to first century CE and have yielded one to several perforated claws [60].

More exceptions can be found in the burial rite itself; it relates to (mostly) inhumation burials with a deposition of unusual bear-parts. Firstly, this relates to skin remains or groups of claws in all corners of a burial that are indicative of decayed skins, as known from little more than a dozen, mainly in Migration Period, burials in Norway and Sweden [58]. All these burials, except one, are above average by their other-than-skin furnishings; however, as a matter of fact, five belong to the richest of their time: four men (three with splendid weapons; graves in Snartemo and Evebø, both Norway, and mound two in Högom in Sweden) and one woman (the so-called ‘petty queen’ from Krosshaug in south-western Norway). Most recently, skins have also been proven by microscopic analysis in a few burials, some wealthy, in Finland, with a dating from 9th to 17th century CE. Secondly, a type of perforated bear-tooth or bear-claw, worn on long straps at the hip or thigh, has come to light in around 40 middle class burials of women and sometimes children from the late fifth to the middle of seventh century CE in ‘linear cemeteries’ (Reihengräberfelder), mostly in the Alamannic area in present-day south-western Germany [55].

The most remarkable finds of bears have not been mentioned so far. These are burials of actual bears known from the northern part of Scandinavia, with a time-depth that covers almost the past two millennia in Norway but only the period post-1000 CE in Sweden [61,62]. A few burials in Sweden are well known and well documented, such as that in Sörviken, Stensele Parish, Lapland, excavated in the 1950s. This burial also yielded, as a rare find, an unfired lead bullet, which belonged to a so-called muzzle loader, a weapon that became more frequent in northern Sweden in the second half of 17th century CE.

Unique for the north are the findings from Frösö, Jämtland, in northern Sweden [63]. Excavations beneath the choir of a medieval stone church brought to light the bones of wild animals, the foremost being bear, together with the stump of a birch tree, surrounded by a dark layer with bones and fire-cracked stones, radiocarbon-dated to the Viking Age. This site has seen intense natural-scientific research and wide-ranging interpretations.

### 3.3. Beaver

**Figure 2:**
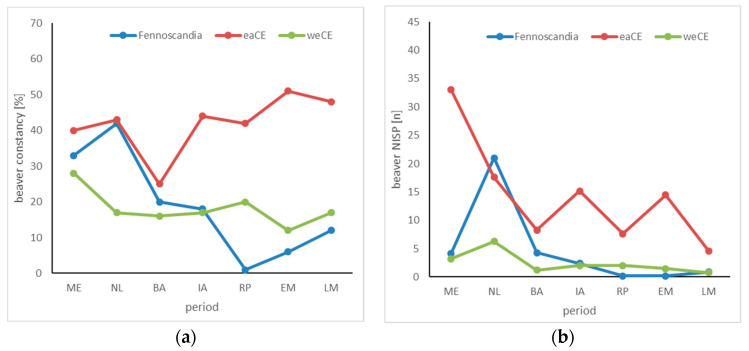
Beaver (*Castor fiber*) at the archaeological site in Fennoscandia, eastern Central Europe (eaCE), and western Central Europe (weCE) in different periods. ME: Mesolithic, NL: Neolithic, BA: Bronze Age, IA: Iron Age, RP: Roman period, EM: early medieval, and LM: late medieval. (**a**) Constancy; (**b**) proportions of the number of identified wild mammal specimens.

Beaver remains are found regularly and in high constancy in most investigated periods of all three study regions (Table 1, Table 2 and Table 3). In FS, the development of the constancy of their remains at archaeological sites is similar to that of the bear. Similarly, the constancy of beaver remains falls off continuously and sharply from the Stone Age (approx. 35%) to Roman times (1%). In the medieval period, beaver remains are found in higher constancy again, but the values no longer reach the level of pre-Christian periods (Figure 2a). The development in eastern CE is the opposite (Figure 2); here, the constancy of beaver remains is generally very high and even tends to increase slightly from the Stone Age (c. 40%) to medieval times (c. 50%). Only during the Bronze Age can a temporary decrease in the constancy of beaver remains be observed.

In western CE, the constancy of the species is very stable from the Neolithic age to the late Middle Ages, with values around 17%. Only in the Mesolithic age are the values higher (28%). Regarding the proportion of beaver in the NISP of remains from wild mammal species, we found striking differences between the three study areas (Figure 2). While the species in western CE never reaches values higher than 6% (at the maximum in the Neolithic age), the values in eastern CE are consistently high, although with a decreasing tendency. While in the Mesolithic age 33% of all wild mammal bones originate from beavers, the proportion decreases to about 12% in later epochs. In the late medieval period, the value even drops to 5%.

The constancy with which beavers can be detected at archaeological sites in eastern CE is even higher. Except for the Bronze Age, it is always over 40% and even increases continuously over time. In the Middle Ages, beavers can be found at every second archaeological site. In FS, the proportion of beaver in the NISP of wild animals was high in the Neolithic age at 21%, but afterwards the value decreases to a constant level, which is only about 2%.

At a few Stone Age sites distributed over all three investigated regions, beaver dominates the refuse fauna, for example, Kaulenkalns (eastern CE, Latvia; Paaver 1965, cited in [64]), Dąbki (eastern CE, Poland [64]), sites in the Dalarna district (FS, Sweden [65]), several locations in south-eastern Finland (in particular, Outokumpu Sätös [66]), and even more in the north [67], or the German sites of Heidmoor (western CE [68]) and Hüde I (western CE [69]).

In all three areas investigated, almost all beaver remains originate from settlement contexts, regardless of the period from which the finds come. Only 4% (western CE), 3% (FS), and less than 1% (eastern CE) of the beaver records derive from graves. However, the large Mesolithic cemetery of Oleniy Ostrov in Karelia mentioned above provides 1201 beaver tooth pendants as grave goods [70]. At that site, beaver teeth were associated more commonly with female graves.

### 3.4. Elk

**Figure 3:**
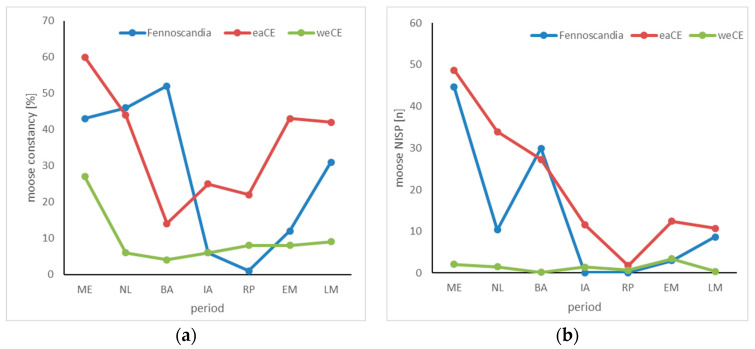
Elk (*Alces alces*) at the archaeological sites in Fennoscandia, eastern Central Europe (eaCE) and western Central Europe (weCE) in different periods. ME: Mesolithic, NL: Neolithic, BA: Bronze Age, IA: Iron Age, RP: Roman period, EM: early medieval, and LM: late medieval. (**a**) Constancy; (**b**) proportions of the number of identified wild mammal specimens.

The archaeozoological record shows that during the Mesolithic age, but also in later periods, elks were a key game species for humans in FS, eastern CE, and also in north-western CE. In eastern CE, elk achieve almost universally high levels of constancy (Table 2; Figure 3a). They are highest in the Mesolithic at 60%, then drop to a low of 14% in the Bronze Age, before rising again almost continuously. At sites from the Middle Ages, elk remains are found again with a constancy of 50–60%. FS follows this development with a time lag. Here, the constancy is also very high in the Stone Age but reaches its maximum value (52%) only in the Bronze Age. Additionally, almost half of the mammal bones found at Mesolithic sites in FS and eastern CE derive from elk (Table 1 and Table 2; Figure 3b). This period also includes special depositions of parts of elk bodies in the shallow water of small lakes after the animals had been slaughtered. Such depositions are known from several sites in Denmark [71,72,73,74] and Sweden [75,76,77]. At the same time, elks are frequently depicted in Scandinavian rock art—often it is the dominant motif—and objects of fine art all around the Baltic Sea [78,79,80,81]. The spread of the elk motif in prehistoric art also reached eastern CE and the north of western CE [82].

In FS, this ‘era of the elk’ is followed by a massive decline in constancy values to a minimum value of only 1% in the centuries around the birth of Christ. From the Iron Age and the Roman period, there were nearly no records of elk at archaeological sites in FS: in the Iron Age, the species was found in one grave and two settlement contexts only—all three in Finland. In the late Middle Ages, elk remains in FS are again found much more constantly (31%). The relative frequency of elk remains in FS in comparison to other wild mammal species is similar to the constancy (Figure 3b). The only significant deviation is the low proportions of elk in the NISP. In eastern CE, the proportion of elk remains in the archaeological record decreases steadily from the Mesolithic (49%) to the Roman period (2%). In the Middle Ages, the values rise again to the level of the Iron Age (by 11%); however, later the size of the population declines in eastern CE. In late medieval times, the distribution of the species was restricted to Latvia and more eastern and northern regions. The situation changed after the beginning of 19th century CE and, by 1810, the distribution of elk in Europe increased greatly and encompasses today’s total FS and the complete area from the Gulf of Finland to the Oder River [83].

In western CE, we see a completely different situation. Here, the constancy with which elk remains are recorded at archaeological sites is low since the Neolithic age never reaches 10%, and is only less than a third (27%) during the Mesolithic age (Table 3; Figure 3a). The proportions of elk remains are even lower and never reach over 3% (Table 3; Figure 3b). An earlier study has shown that elks in Western Germany up to the river Elbe have only been occurring in very low population densities since the Neolithic age [84]. From the Bronze Age to medieval times, we see here two distinct populations, the first in the triangle of the Harz mountains in the north, the Thuringian Forest in the southwest, and the Ore mountains in the southeast, and the second being a population along the Elbe River, which likely had a fairly high population density. Both populations became extinct at 1200 CE at the latest. Following the archaeological records, in the eastern part of western CE, the elk population was much larger; here, the species is more often identified at archaeological sites from the Stone Age up to early medieval times. But again, also in this area, the youngest record dates from approximately 1200 CE. In the north of western CE, at Mesolithic sites, elks can be recorded with high constancy, but after the ‘neolithisation’ about 4000 BCE, records become very rare [84]. Only in the hinterland of the southern coast of the Baltic Sea, do the species survive much longer. However, as in the other parts of western CE, it was last mentioned in 13th century CE [85].

## 4. Discussion

### 4.1. Birds of Prey

The special human connection with birds of prey can be traced back to the era of the Neanderthals [86]. However, in the area of interest in the present paper, clear evidence for their symbolical or ritual meaning during Mesolithic and Neolithic times is largely missing. The only exception is the aforementioned burial finds of osprey on the island of Oleniy. These are obviously something special, as ospreys were common throughout the study area at the time [84]; however, this can only be proven in these particular grave contexts. Accordingly, one should be careful with generalizing interpretations; perhaps for some people, ospreys were mediators between the human and the spiritual world, which accompanied the soul to the afterlife [24,86]. In contrast, it seems that white-tailed eagle, by far the most frequently recorded raptor species at Stone Age sites, were hunted particularly because of their large feathers, but perhaps also for bone artefacts or their meat. During the winter, white-tailed eagles are easy to kill with a bow and arrow when they gather around the last ice-free patches of frozen water. Therefore, it is not very likely that shooting such an animal would have greatly added to the status of the hunter, as was suspected [87]. Probably, it is rather the partly very high constancy of the species in archaeozoological assemblages that underlines the special economic importance of that mighty predatory bird—a similar conclusion has been drawn for the Netherlands [87,88].

The starting point for any further discussion of raptor bones in burials, settlements, and imagery is the fact that these birds—mainly goshawks—are not attracted to humans [89,90]. Thus, we observe here an extraordinary animal–human relationship. An explanation for this phenomenon can be found in two ways: firstly, an interdisciplinary look at sources from premodern times for disciplines other than archaeo(zoo)logy and, secondly, by taking into account present-day knowledge, that is, thus, transdisciplinary.

The so-called Germanic Law Codes—the oldest are c. 1500 years old—for tribes in parts of Central Europe that point to hunting with trained birds of prey, are more or less contemporary with the aforementioned burials, such as the one in Quedlinburg [91,92]. Furthermore, there are literary sources for the North—Scaldic Poetry and Saga Literature—which also refer to this kind of hunting [93,94]. Written down in Iceland from 13th century CE onwards, the reported narrations may go back to the late first millennium CE, if not to older times.

It is only by the period post-1000 CE that pieces of evidence become more numerous and informative. A central place is held by Emperor Frederick II’s book ‘*de arte venandi cum avibus*’ (on the art of hunting with birds) from early 1240 CE [95,96]. This entire book, written after decades of practical experience, is about falconry and is still considered as very valuable for all the details about its practical aspects (personal communication, Karl-Heinz Gersmann). This book also reflects a certain philosophical thinking on the period in question, since only the ruler that could control his/her temper (and thus was a good falconer) was also a good emperor. In addition, to provide examples from more recent times, the paintings of Rembrandt van Rijn and theatrical plays by William Shakespeare can only be understood in the context of falconry [97,98]. Worth a mention are also the extensive records on Maria, Duchess of Burgundy (1457–1472), a prime representative of noble (royal) falconry and hunting practised by women [99].

From today’s viewpoint, the raptor species themselves (mainly goshawks) point to the practice of falconry, which is defined by modern practitioners as follows: taking quarry in its natural state and habitat by means of a trained bird of prey (definition by the International Association for Falconry and the Conservation of Birds of Prey; www.iaf.org, accessed on 26 January 2024) Since these birds are not attracted to humans, it is the task of the latter to try to build up a relationship with the bird, the first step being when food is offered until this finds acceptance by the bird [89]. All that can be hoped for is comradeship, as once defined by Konrad Lorenz, which is not domestication [100]. To have this relationship with a bird means daily contact in a strict regime, and hunting is only part of that. When it comes to the hunt itself, the falconer says, quite remarkably, it is not the human who goes hunting with the bird, it is the other way around. And this hunting is observed with mixed emotions: admiration of the bird’s aerial manoeuvres, on the one hand, but also fear that the bird may not return [89].

It is only with this knowledge that archaeological finds can be properly understood. Ideally, falconry is indicated via the presence of the remains of both the trained birds and their typical prey, plus falconry equipment. Regularly, with the Rickeby burial as a prime example, the goshawk (but also the sparrow-hawk and peregrine falcon) are recorded as birds used in falconry but so are typical prey, such as black and hazel grouse [27,29]. It is also of importance that goshawks were preferred, as dwellers and hunters of the forest, in areas that were much more forested than today. Only against this broader background can falconry equipment be identified, such as small metal bells (at the bird’s feet, so it can be followed acoustically), gloves (protection of the falconer’s hand against the sharp talons), and hoods (for the falcons, so stress can be reduced before the hunt). Suffice to say here that only bells have to come light in some numbers, and one particular type that is well-known from Central European strongholds post-1300 CE can be ascribed a use in falconry; they can also be compared to modern falconry bells [39,101].

Falconry, as it was described above, was a leisure activity of a social upper class right from the start, judging from archaeology but also written accounts [13,14]. But beware: historically, falconers were the practitioners who took care of the birds on a daily basis, whereas the noble (royal) persons were barely interested in hunting. Once a bird had become accustomed to a falconer, it could be placed on the fist of another person as well.

It also should not go unnoticed that falconry may have been invented in the Eurasian Steppe, for practical reasons (fur acquisition; protection of flocks), with the use of Golden Eagles. As one may argue, after it had been invented in the steppe, the knowledge of falconry spread to a stretch of land that reached to the remote east (Japan), remote west (Spain and Portugal) and remote South (India and Arabia) [14,102]. According to the falconer, however, it is only a small step from observing the raptor pursuing prey in nature towards the idea of becoming a part of it. The actual making of falconry equipment would follow quite naturally, as form follows function, and thus one may observe here a case of multiple rather than single invention [103].

Finally, it bears mentioning that during medieval times, large birds of prey, mostly white-tailed eagles, were sometimes found in large quantities, e.g., in Hedeby (9th to 11th century CE [50]) or Klaipėda (13th to 14th century CE [51,104]). In both cases, these birds were probably killed in order to use their feathers, whereas a meaning of the birds in spiritual life or as status symbol cannot be established (cf. [105]).

### 4.2. Brown Bear

Humans have always a very special relationship with the brown bear. After all, the species is not only the largest land predator in Europe but it is also the animal that has the greatest similarities to humans: the faces of bears look human-like from the front and bears show remarkably intelligent behaviour: they build their living quarters themselves; they can swim, climb trees, and stand on their hind legs; they can use their front paws to grasp and carry; and they are omnivores like humans [106]. The knowledge of such behaviours gives rise to numerous and widely spread legends of humans being reared by bears or experiencing shape-shifting into a bear [107,108]. The special relationship between the bear and human finds reflection, inter alia, in so-called bear ceremonials and taboo name giving.

Evidence of a special relationship between humans and bears dates back to the Late Palaeolithic age. Bones of the cave bear (*Ursus spelaeus*) from several Belgian caves show traces of red ochre, which was deliberately applied by humans [109]. The skull and the bones of the paws are particularly affected. Since such colouring of the bones has no immediate use, it can be viewed as an indication of ritual behaviour. Remarkably, it is still common in some Nordic societies to colour the head/skull and paws of killed bears during special rituals [109].

Regarding western CE, it is remarkable that during the Mesolithic age—i.e., during the only period when human life was based exclusively on the exploitation of natural resources—the relative NISP of bear remains reaches its all-time minimum. This result finds its parallel in Austria, where bears are completely lacking during the Mesolithic age [110]. At the same time, however, bears were equally present throughout the complete study area. The rarity of bear bones does not stand alone, since other Carnivora are also recorded only rarely at Mesolithic sites and mostly with only a few bones per site [12]. This leads to the conclusion that species such as the bear (or wolf) were not a regular hunting prey in Mesolithic times in large parts of central and northern Europe [111]. Even the fact that artificially perforated bear canines have been repeatedly found in the area of investigation is not a contradiction. Such bear canines were part of the general and common practice of using the teeth of large animals as clothing accessories [112]. It is likely that spiritual reasons prevented the persecution of bears or limited it to exceptional cases. Presumably, the killing of a bear was only allowed when it involved the performance of complex rituals before and after the hunt.

Bear ceremonialism is well documented in FS for the Sámi, Finns, and Karelians, based on rather recent records, i.e., reports of missionaries, post 1500 CE, for the Sámi and the recording of so-called bear songs for Finns and Karelians in the 19th century. The ceremony used to be a set of rules that was meant to guarantee the respectful treatment of a killed bear so that it would report this to the assembly of bears in the otherworld [113,114,115,116]. The ceremony of the Sámi consisted of three elements: the bear hunt, bear feast, and bear grave, whereas the ceremony of the Finns and Karelians consisted of a bear hunt, bear feast, and bear skull tree. With this knowledge, the aforementioned bear graves in the northernmost Scandinavia are immediately recognisable as the last element of Sámi bear ceremonialism, whereas in the case of the Finns and Karelians, traditions are still attached to certain trees into which branches of bear skulls were placed. How old these ceremonies really are and if all of them, despite their differences, go back to one common source (see here, the rightly famous [117]) is open to debate; however, the mere existence of such ceremonies points to a population for which hunting continued to play a vital role, as opposed to farmers who increasingly, as one may assume, saw bears as mere pests [58,117].

When it comes to the north Germanic population of FS, there are no records about bear ceremonialism, but the ancient taboo of name giving, and the aforementioned particularly late (just before the advent of Christianity) Viking Age Fröso finds spot, may be considered as indications for this. The present term ‘bear’ means ‘the brown one’, with reference to the colour of the skin ([118]; ‘honey-eater’ is a taboo word used in Slavic languages [119]). It is argued that the real name for the bear had to be avoided. It was feared that the animal could overhear human communication and that this could conjure up an unpleasant encounter. Again, as was the case with bear ceremonies, an advanced age could be taken as a given for the taboo name, as a reflection of a population for which hunting was still important, whereas it was different for a society of mere farmers. The aforementioned Frösö site, with its highly impressive archaeo(zoo)logical findings, may be reminiscent of a local bear feast and the deposition of bear remains at the foot of a tree, as an expression of some kind of a ritual [63]. Notably, the very place name itself, Frösö, alludes to a god of the old faith and suggests a place of a cult.

The actual bear hunt is also telling. For the Sámi, hunting at the bear den has been recorded up until recent times; it was a communal activity that was meant to keep the danger low for the hunters. However, there is also imagery that shows fights with bears [61]. In contrast, we know of bear hunting, in Roman and Germanic contexts, as a ‘heroic deed’, with the animal driven by dogs to a desired place where the hunter would wait with a bear spear [108,117]. Furthermore, the late antique scribe, Ammianus Marcellinus, left a somewhat distorted description of an otherwise unknown Germanic tribe that may point to the dispatching of a bear as initiation rite for reaching adulthood or for being admitted to the warrior community. One is tempted to think that, in particular, the aforementioned wealthy weapon burials of men with actual skin remains has a place in this tradition; were the deceased also staged as ‘heroic hunters’? However, such skins were found in burials of women, too, including the aforementioned ‘petty queen’; the question has to be raised about the ideological background for these.

### 4.3. Beaver

For many millennia, beavers must have been among the most enigmatic creatures in human habitats: they live in water and have a scaly tail but are not fish; they come ashore at night and cut down trees; and they can change entire landscapes and cause floods. Our results clearly show that beavers were present in archaeological assemblages in all three regions over the studied periods, regardless of climate and cultural changes. Beyond that, however, differences predominate. Animal remains from Mesolithic and early Neolithic sites in northern Sweden show beaver next to elk as the most hunted mammalian species (Figure 2 and Figure 3; cf. [65,120]), and we can be sure that the animal was hunted for its meat, fur, and maybe its castoreum. Beaver meat is a high-quality protein source due to its well-balanced essential amino acid composition [121], and it is also a valuable fat resource, since a beaver carcass contains 15% fat [122]—a value significantly higher than in all other non-marine species in the region.

At least in the easternmost parts of FS and eastern CE, they also had a distinctive role in human burial rituals. At the Karelian Mesolithic burial site of Oleniy Ostrov, for instance, beaver teeth seem to have been connected with the age, sex, and social status of the deceased [123,124,125], and at Zvejnieki, a Mesolithic cemetery in Latvia, bone pendants made of beaver astragali were found in several graves [126]. Although this finding is unique in this form in the Mesolithic study area, it shows that, at least regionally, there must have been a religious meaning or spiritual association with beavers.

The data from western CE show the least changes over thousands of years. Here, beaver hunting never had a high intensity and was quantitatively insignificant (see Figure 2b). At the same time, the constancy of their records at archaeological sites of just under 20% over long periods is evidence of stable populations (Figure 2a). In FE, on the other hand, the number of finds and the relative frequency of records declined with the onset of the Metal Age. Since beavers undoubtedly continued to live everywhere in FS in those days, it must be assumed that beavers were deliberately hunted here far less than other species. It was not until the early medieval period that the constancy values increased again, perhaps in connection with the supra-regional fur trade. The increasing constancy in the late medieval period finds its parallel in western CE. In both cases, it indicates the beginning of the targeted persecution of the beaver, which ultimately led to the disappearance of the species because of overhunting in large parts of western CE and FE in the following centuries. In FS, the last wild beavers were shot in the 19th century, with only a small population surviving in southern Norway, which later served as a place for various reintroduction projects [127]. After they had been placed under legal protection and targeted conservation measures were started, beavers have been able to spread over large areas of FS since the second half of the 20th century, developing large and increasing populations again [128]. As already mentioned, our data also show an increase in the constancy for western CE in the late medieval period, which coincides with the onset of hunting aimed at extermination. However, the development in western CE must be viewed in a differentiated manner. In the north, in Denmark, beavers disappeared as early as the Bronze Age, but at the very latest in the Iron Age around 500 BC [129]. Their disappearance coincides with a period of intensified agricultural exploitation of the landscape, leading to the formation of the first heathlands [130]. Intensified farming and agriculture could easily result in conflicts of interest between the ‘landscape gardener’ beaver and humans in their shared habitat along rivers and streams. Populations also declined rapidly further south, and Gesner (1581) mentions only the Elbe and Saale as areas in western CE with significant beaver populations in Germany.

In eastern CE, we observe a different development. Here, beavers remain a quantitatively important hunting prey even in the Metal Age, accounting, on average, for about 10% of all mammal bones from archaeological contexts, and the constancy with which beavers are detected in archaeological sites is usually over 40%. In Roman times and the early medieval period, perforated astragali from beavers were popular in the east of eastern CE [131]. They are found as decorations for the deceased, but also seem to have been used to represent a person’s social status during their lifetime; meanwhile, in Estonia, for example, all known beaver astragali pendants come from settlement contexts [131]. Further east, necklaces made of beaver astragali were found exclusively in women’s graves [11]. The metaphysical or cultural background of the pendants made of beaver bones remains speculative, although astragali played a very special role in antiquity and were used as oracles and amulets [132,133,134]. In the case of beaver astragali, interpretations range from the assumption that such applications identified fur trappers or traders and their wives [135], to the assumption of a cult associated with beavers [11], to a status symbol reflecting the very prominent economic importance of the species [131]. Nevertheless, amulets made from animal products were likely intended to transfer characteristics of the animal to humans, be it vitality, strength, or power [131]. It should be noted that at the same time, in England, pendants made of beaver teeth have been found exclusively in graves of babies, children, and juvenile or adult females, often decorated with a pierced metal cap made of copper alloy, bronze, gilt bronze, or even gold [136]. As in eastern CE, there is a strong connection to children or (pregnant) women. In England, interpretations focus on the beaver’s homebuilder qualities, or they point to the similarity of the Latin words castor for beaver and casitas for chastity; in this context, the beaver remains could refer to the virginity of the deceased [137]. The association of beavers with virginity may stem from the fact that humans have probably almost never observed beavers mating. The mating takes place in the coldest winter, at night, in the water. Moreover, beavers are monogamous and form lifelong relationships to their partner. They live in families that usually consist of the parents and the last two generations of young animals.

This group life may be the root of the following aspect: Wade [138] recently pointed out another and completely different aspect of the human view of beavers during the medieval period. He shows that various contemporary sources from the 12th century, in between the *Historia Norwegiae*, describe beaver populations as ‘slaveholding societies’, implying that slavery is a natural phenomenon. Free and enslaved beavers can, according to the sources, be distinguished by, for example, the quality of the fur. The age and origin of this narration is unknown but the most detailed versions are those from Norway and England [138].

The background to the close human–beaver connection might not be easy to understand, but a modern example shows how beavers can have a high level of importance in the worldview of social groups. In the case of the Mistassini Cree hunters living on the Labrador Peninsula, Canada, not only the creation myth is fundamentally connected with beavers but, in their whole ideology, the beaver is central [139,140,141]. Nevertheless, the Mistassini Cree do hunt beavers, and especially during winter, both their rituals and food economy focus strongly on these animals. Also, from FS, it is reported that people perceived beavers as people with different strengths and abilities. Here too, certain rituals had to be completed before hunting and killing and also afterwards [142,143]. They served to ensure the rebirth and new life of the hunted beavers.

For people with forager lifeways, cohabitation with beavers improved their habitat and their living conditions. In many cases, it was the beavers’ agency that created suitable settlement places and pathways in the forested landscape [120]. In North America, this resulted in a widespread aversion to beaver hunting and in a role and standing of beavers in a belief system quite similar to the status of cattle in India today [144]. The more common and widespread beavers were in a region, the clearer and more natural the importance of the ‘landscape gardener’ beaver seems to have been to hunter–gatherer–fisher communities. Thus, following Hussain and Brusgaard [120], beavers were an ecological keystone species for humans with a ‘Mesolithic’ lifestyle, but in the course of the very long joint history of human–beaver co-habitation, the beaver also became a ‘cultural keystone species’. The extremely careful handling of tools made from beaver lower jaws by Mesolithic humans [145] could also be an indication of the ideal significance that even tools made from beaver bones had.

Additionally, the fact that a metonym was necessary for the beaver as a result of a naming taboo shows that it was an extraordinary creature for prehistoric humans. Etymologically, ‘beaver’ has the same origin as ‘bear’ [11]: it is a duplication of the syllables *bher- or *bheru-(‘The Brown’). This origin of the animal’s name is widespread, and also visible in, e.g., the Lithuanian ‘bēbras‘, the Dutch ‘bever’, the German ‘Biber’, and the Czech bobr‘ [146,147]. Because of all the special beaver characteristics—not to mention the nocturnal sounds of beavers gnawing and felling in the forest—it is plausible to see here the root of the legend of humanoid water spirits known in many European cultures (Vodyanoy, Hastrman, Aquarius, etc.) [11].

It should also be mentioned that, due to the high demand for beaver fur in large parts of Western and Central Europe, real breeding stations for beavers emerged in Poland in the late Middle Ages [148]. So many beaver bones were also found at individual settlement sites in Estonia and Latvia that, at the least, a specialized management of beavers must be considered [148].

Despite several different approaches, in the case of the beaver, diachronic cultural-historical, philological, and ethnological studies on this special human–animal relationship are still in their infancy [11,120,149,150]. This s a valuable perspective for future work to test the hypothesis of Hussain and Brusgaard [120], in which human–beaver systems can be described as ‘commensal’—with human foragers being commensal *to beavers*, since it was finally the beavers’ activity that turned former woodland into flooded areas as well as into meadows with feeding opportunities for hunting prey (deer species; wild boar) and human livestock.

### 4.4. Elk

Elks, Europe’s largest deer species, live sociably in small groups in cool forests and on forest edges, usually near bodies of water. The males have large antlers, which they shed yearly, and they display impressive, loud, aggressive rutting behaviour with sparring matches in autumn. Due to their large size, widespread distribution, abundance, and noticeable behaviour, elk are the characteristic species of the forested part of northern Europe. It should come as no surprise that they were among the most important wild animals for prehistoric humans there, not only as suppliers of meat, antlers, bones, tendons, and fur but far beyond that [65]. According to V. Mantere’s thorough work, which is worth reading, there were two main reasons why the elk, of all animals, acquired a high symbolic and ritual significance [81]: the high efficacy of elk hunting, and the versatility of the elk as a resource. Being a solitary and dangerous species, hunting elk requires the individual to be skilled and experienced [65,76].

A closer look at the Mesolithic age shows that elk had its major significance as human-hunted prey in FS and eastern CE between 9200 and 7000 BCE [65]. This was also the period of the first elk depictions in Scandinavian rock art, and all rock art studies show a complex relationship between humans and elks that is comparable to that with bears. The recurring and often similar elk motifs in the whole FS suggest very similar fundamental conceptions and beliefs all across the region [78], and it seems that the conceptions were in different ways connected to ideas of the other world [151]. Based on archaeological and ethnohistorical studies, Bolin [78] sees indications that the Mesolithic and Neolithic people in FS believed in a mythical elk that was once the ancestor of mankind. This belief was transmitted in the elk–boat metaphor often observed in rock paintings [152]. The high number of rock art depictions [153,154] and elk-related artworks [77] in eastern CE and FS demonstrate, in any case, the species’ central role in the spiritual concepts of the hunter–gatherers. Such artworks are so numerous that the elk’s importance in the world view of the prehistoric people of Northern Europe can hardly be overestimated. There are still many uncertainties regarding the meaning of artwork depicting elk—and so of the general position of elk in the ‘plurispecies’ society (see below) in those days—, but it seems that they were closely connected to power and prestige and demonstrated the potency and special status of their owners, who were always mature and mostly male [155]. However, a detailed analysis of all available sources shows that it was the elk cow that was quite obviously the centre of attention [65]. Elks were so important for human survival in the Mesolithic age in FE, eastern CE, and the north of western CE that it was highly necessary to ensure their successful reproduction. Against this background, female elks, but also female deer, in general, were observed as givers of life with ultimate and general control over rebirth and fertility, human hunting success, and, ultimately, life and death [156].

Mesolithic artworks were the continuation of older traditions, since the elk already formed a focal element of final Palaeolithic art in the whole study area, i.e., the inclusive western CE [157]. From the early Mesolithic age of Denmark (northernmost western CE) and Scania, Sweden (southern FS), depositions of selected elk and sacrifices of hunted elks are known [65,73,77]. The depositions of parts of slaughtered elks on the shores of small Danish and Swedish lakes, however, likely had no ritual character but were used to preserve excess meat in cold water. Nevertheless, they indicate that hunted elks were processed according to uniform practices [74].

In that region, i.e., the modern western Baltic Sea area (in those days, the Baltic Sea did not exist), but also in all of western CE, the relative NISP of elk decreases during the Mesolithic age. Presumably, the elk population size has now dropped drastically due to severe climate warming, and the species may have even started to disappear from some areas [82,84]. But although elks are now becoming much rarer, they remained very special creatures to humans [151]. This is particularly evident in the phenomenon of schematic elk heads from several archaeological sites in northern western CE, which were sculpted from red deer antlers [82].

For the Neolithic age, our results show a sharp decline in the NISP—but not in the constancy. Also in FS, this is a phenomenon that has been observed by other researchers before and dates to a late stage of the northern Scandinavian Neolithic age (the centuries after 2200 BCE [77]). It is likely that the decline is linked to a drastic change towards a colder and wetter climate (in a noticeable contrast to that, in western CE, it was the climate warming that limits the elk’s occurrence), and as a consequence of this climate development, the elk population became smaller. As a consequence, the species also disappeared step by step from the human ritual sphere [77], but as the still high constancy values during the Mesolithic age and Bronze Age show, this was a slow process.

In the Iron Age and during the Roman period in FS, we observe an almost complete absence of elk in archaeological finds. At least regionally, the species was still present at that time in FS, but their populations were relatively small during the Iron Age [157]. Apparently, in FS, elk bones were not left at the settlements after the meal as waste in the centuries around the birth of Christ, and this custom is also indicated by ethnographical sources, which show that elk (and other cervid) bones were often handled ritually [158].

In the subsequent early Middle Ages from 800 to 1300 CE, elk still played a role in Finnish death rituals, especially in the burials of women, and there are references in epic poetry to elks transforming into women (Siikala, cited in [159]). Such a human–elk transformation is also discussed in the case of the so-called ‘Elk-man’ buried under the threshold of a Viking Age longhouse in Birka, Sweden, with a complete, unornamented elk antler by his head and a decapitated man as companion [160,161]. Since this feature is absolutely unique, it is hard to understand the background, in particular regarding the role of the elk antler. All interpretations remain highly speculative.

It is evident both in the decreasing amount of archaeological and archaeozoological evidence, and in the small number of (more recent) myths and fairy tales handed down, that the elk lost its important position within the system of belief earlier than the bear [65,161,162]. Presumably, this loss of lore is due to the disappearance of the species not only from western CE and eastern CE but also from FS in the course of the second millennium CE. The few myths and fairy tales about the ‘cosmic’ elk depict it in an elusive, ambiguous way, with its home in the arch of the sky linked to the land of the dead [162]. Since some hunting myths also survived, the elk was likely also at the centre of an ancient hunting cult [162].

## 5. Conclusions

All the animal species discussed here clearly had meanings for humans that went far beyond that of a mere resource. However, the archaeological record also shows differences that may indicate which particular species had significance in the context of death and burial. At least in certain regions and at certain times, the remains of bears and birds of prey originate regularly from human graves, and with birds also originating from ‘special settlement sites’, such as seats of power. Beaver and elk, in their turn, are rather rare in a funerary context.

Among the considered wild species, raptors had a place of their own as companions of humans (K. Lorenz). It was the human who had to try to establish contact with the bird and find its acceptance in what would be a fragile relationship. The advent of Christianity had no influence, except for the fact that birds were no longer added to human burials. It was the period post-1000 that saw the real blossoming of falconry as a courtly art, now with falcons and from horseback, and it even turned into a matter of state philosophy in the period of Holy Roman Emperor Frederick II in the early 13th century—only the ruler who could control his/her temper, like a falconer, was also a good ruler.

Bears also had a special position when considered together with the beaver and elk, since they were almost like ‘humans in disguise’ (due to the similarities with humans) and were a tabooed being whose name had to be avoided. In the past, hunting wild animals such as elk, beaver, or bear required a well-founded knowledge of their complex behaviours and ecological requirements. Even to some degree in recent times, but certainly in (pre)historical societies, such knowledge was associated with beliefs and ideas about animal personhood, agency, and multi-layered human–animal relationships. As a consequence, the foragers saw themselves not as the ‘crown of creation’ but as part of a ‘plurispecies’ society with many equally ranked subjects [120,163]. To these societies in the Mesolithic woodlands of northern Europe, it was not the human but the bear, beaver, and elk that were central (cf. [120]). There can be no doubt that three out of four species considered in this contribution played a fundamental role for people in northern Europe and their perception of nature. They were associated with existential considerations that went far beyond profane food–economic necessities. This ‘plurispecific’ world view persisted the longest and was the most pronounced in eastern CE, and for at least as long in western CE.

The decline and ultimately the disappearance of the animal symbolism described here and with it the disappearance of an invisible but strong bond between humans and animals, but also the end of a worldview of a ‘plurispecies’ society in general, had different causes depending on the region and time. They were natural or had their roots in social transformation processes. Climate changes drove elks out of large parts of their original range and bears withdrew due to habitat fragmentation [21,120]. In other cases, it was cultural transformations that led to changes in beliefs and the understanding of the world. These transformations include ‘cattle-ization’ [164], which spread to large parts of Europe with the beginning of livestock farming, or the change from the pagan to the Christian faith, which led to profound changes in the relationship between humans and animals. After Christianisation, the contrast between humans, on the one hand, and animals, on the other, began to change the worldview. Difference was constructed, and the animal became ‘the Other’. The millennia-old permeability and blurring between the concepts of ‘animal’ and ‘human’, which we encountered repeatedly in this paper, disappeared in favour of a strict dichotomy. However, besides Christianity, the demotion of animals in the philosophy of Classical Antiquity and the Period of Enlightenment must not be overlooked.

Following a multispecies approach, human–animal interactions should avoid the modern antagonism between animals and humans and see bears, beavers, elks, and all other animals as equal. Only when the modern anthropocentric perspective is overcome is it possible to gain access to the human understanding of ‘animals’ that was established in northern Europe millennia ago. Opposites then fade away and mutual similarities and commonalities come to the fore instead. In this way, elks, bears, and beavers become Alter Egos: beings like humans, just in a different form. It is an integral component of this perspective that animals have rights—including, in (pre)historical thinking, the notion of life after death and rebirth—and also a dignity that must be preserved when hunting, killing, and using them. The assumption that humans (at least in our study area) saw themselves as part of a ‘plurispecies’ society over long stretches of their history is much more suited to archaeo(zoo)logical findings and thus comes much closer to the ‘truth’ than interpretations that assume people of earlier epochs would have followed a kind of ‘optimal foraging theory’, a utility-oriented decision-making. Without any doubt, the processes behind prey selection, and the decision to kill a particular animal, were much more complex than that. Especially for the bear, but also for beaver and elk, there was a ceremony before and after hunting and killing.

## Figures and Tables

**Table 1:** Fennoscandia. The NISP is only mentioned for wild animal species. CON: constancy. The few finds from Late Glacial (only a total NISP of 48) and early modern times (only 3 sites) are not meaningful and have been excluded.

		Elk	Bear	Beaver		Elk	Bear	Beaver
Period	Sites (n)	CON (%)			NISP ^1^	NISP (%)		
Mesolithic age	108	43	17	33	19,345	44.6	0.9	4.2
Neolithic age	103	46	17	42	29,840	10.4	0.2	21.0
Bronze Age	25	52	4	20	1459	29.9	0.1	4.3
Iron Age	49	6	31	18	4225	0.1	0.9	2.4
Roman Period	71	1	82	1	1001	0.1	5.8	0.2
Early medieval period	50	12	12	6	1208	3.0	0.5	0.2
Late medieval period	59	31	12	12	2787	8.7	0.4	0.9
	465				59,865			

^1^ wild mammal species only.

**Table 2:** Eastern Northern Central Europe (eaCE). The NISP is only mentioned for wild animal species. CON: constancy.

		Elk	Bear	Beaver		Elk	Bear	Beaver
Period	Sites (n)	CON (%)			NISP ^1^	NISP (%)		
Mesolithic age	10	60	40	40	2041	48.7	2.2	33.0
Neolithic age	118	44	31	43	75,115	33.9	2.1	17.6
Bronze Age	87	14	18	25	13,089	27.3	2.3	8.3
Iron Age	64	25	16	44	7196	11.6	1.6	15.2
Roman Period	41	22	10	42	3569	1.8	4.0	7.6
Early medieval period	89	43	45	51	11,381	12.4	1.7	14.5
Late medieval period	150	42	39	48	40,371	10.7	1.4	4.6
Early Modern period	19	21	21	21	1521	18.6	2.5	2.1
	578				154,283			

^1^ wild mammal species only.

**Table 3:** Western Central Europe (weCE). The NISP is only mentioned for wild animal species. CON: constancy. The few finds from Late Glacial (only a total NISP of 48) and early modern times (only 3 sites) are not meaningful and have been excluded.

		Elk	Bear	Beaver		Elk	Bear	Beaver
Period	Sites (n)	CON (%)			NISP ^1^	NISP (%)		
Mesolithic age	159	27	18	28	24,408	2.1	0.2	3.2
Neolithic age	409	6	12	17	56,900	1.5	1.0	6.3
Bronze Age	90	4	18	16	12,070	0.2	0.8	1.2
Iron Age	135	6	14	17	10,388	1.4	1.6	2.0
Roman Period	261	8	17	20	18,933	0.7	1.6	2.0
Early medieval period	252	8	9	12	9655	3.4	0.8	1.5
Late medieval period	205	9	21	17	28,291	0.4	1.0	0.8
	1511				160,645			

^1^ wild mammal species only.

**Table 4:** Eastern–Northern–Central Europe (eaCE). Constancy of birds of prey in different periods of time. Others include the remains of birds of prey identified to genus level only as well as western marsh harrier (*Circus aeruginosus*), hen harrier (*Circus cyaneus*), osprey (*Pandion haliaetus*), rough-legged buzzard (*Buteo lagopus*), Eurasian hobby (*Falco subbuteo*), merlin (*Falco columbarius*), and saker falcon (*Falco cherrug)*.

	Sites (n)	Others	Common Buzzard	Golden Eagle	Gyrfalcon	Peregrine Falcon	Common Kestrel	Sparrow-Hawk	Goshawk	White-Tailed Eagle
Mesolithic age	10	10.0	10.0	20.0						20.0
Neolithic age	118	3.4							1.7	4.2
Bronze Age	87	1.1	1.1	1.1			2.3		1.1	3.4
Iron Age	64			3.1					1.6	3.1
Roman Period	41		2.4	2.4			2.4		2.4	
Early medieval period	89	1.1				1.1	1.1	1.1	6.7	3.4
Late medieval period	150	3.3	3.3	1.3	0.7	0.7	2.7	3.3	14.0	8.7
	559									

**Table 5:** Western CE: Constancy of birds of prey. Others include the remains of birds of prey identified to genus level only as well as western marsh harrier (*Circus aeruginosus*), hen harrier (*Circus cyaneus*), Montagu’s harrier (*Circus pygargus*), osprey (*Pandion haliaetus*), greater spotted eagle (*Clanga clanga*), lesser spotted eagle (*Clanga pomarina*), black kite (*Milvus migrans*), rough-legged buzzard (*Buteo lagopus*), Eleonora’s falcon (*Falco eleonorae*), Eurasian hobby (*Falco subbuteo*), and merlin (*Falco columbarius*). From gyrfalcon (*Falco rusticolus*), there are no records known.

	Sites (n)	Others	Common Buzzard	Golden Eagle	Red Kite	Peregrine Falcon	Common Kestrel	Sparrow-Hawk	Goshawk	White-Tailed Eagle
Mesolithic age	159	7.5	4.4		1.9		0.6	0.6	0.6	23.3
Neolithic age	409	1.2	1.0	0.8	0.3	0.3		0.5	1.2	3.4
Bronze Age	90	2.2				1.1	1.1		2.2	1.1
Iron Age	135	1.5	1.5	3.0	2.2		1.5		4.4	3.0
Roman Period	261	2.3	1.5	0.4	0.8		0.4	0.8	3.5	5.4
Early medieval period	252	3.6	2.0	1.2	1.2	1.2	0.8	2.4	4.4	5.6
Late medieval period	205	4.9	3.4	1.0	2.4	1.5	1.5	4.9	10.7	8.8
	1511									

**Table 6:** Fennoscandia. Constancy of birds of prey in different periods of time. Others include the remains of birds of prey identified to genus level only as well as western marsh harrier (*Circus aeruginosus*), hen harrier (*Circus cyaneus*), Montagu’s harrier (*Circus pygargus*), osprey (*Pandion haliaetus*), rough-legged buzzard (*Buteo lagopus*), Eurasian hobby (*Falco subbuteo*), and merlin (*Falco columbarius*).

	Sites (n)	Others	Common Buzzard	Golden Eagle	Gyrfalcon	Peregrine Falcon	Common Kestrel	Sparrow-Hawk	Goshawk	White-Tailed Eagle
Mesolithic age	108	0.9	1.8	2.7	0.9		09	0.9	2.7	4.6
Neolithic age	103			1.0				1.0	1.0	2.9
Bronze Age	25							4.0		4.0
Iron Age	49									2.0
Roman Period	71	7.0	1.4				2.8	2.8	4.2	4.2
Early medieval period	50	12.0		2.0	6.0	14.0	2.0	10.0	72.0	6.0
Late medieval period	59	16.9	3.4		3.4	5.1	3.4	6.8	25.4	27.1
	465									

## Data Availability

The archaeozoological data for this study are gathered from the huge data collection “The Holocene History of the European Vertebrate Fauna” (https://doi.org/10.13149/001.mcus7z-2). This data collection is Open Access available at http://datenportal.ianus-fdz.de/pages/collectionView.jsp?dipId=1650048 (accessed on 26 January 2024).
